# Acute Hepatitis A With Polyserositis and Acalculous Cholecystitis in an Adult: A Case Report

**DOI:** 10.7759/cureus.95901

**Published:** 2025-11-01

**Authors:** Syed Faraz Abbas, Vaibhav Shukla, Saboor Mateen, Firdaus Jabeen, Ajay Singh Yadav

**Affiliations:** 1 Internal Medicine, Era's Lucknow Medical College and Hospital, Lucknow, IND

**Keywords:** acalculous cholecystitis, acute hepatitis, ascites, hepatitis a, pleural effusion

## Abstract

Hepatitis A virus (HAV) infection in adults is usually self-limited, yet atypical extrahepatic features may complicate the course. We report a 20-year-old male with fever, jaundice, and moderate ascites. Evaluation showed marked hepatocellular injury with direct-predominant hyperbilirubinemia and positive HAV IgM. Imaging demonstrated hepatosplenomegaly, bilateral pleural effusions, and gallbladder wall edema without calculi, consistent with acalculous cholecystitis; there was no encephalopathy. He received conservative management. After initial improvement, a transient mid-course bilirubin rebound occurred, compatible with self-limited intrahepatic cholestasis, followed by steady recovery. By three months, liver tests had normalized; HAV IgM remained weakly positive, and HAV IgG seroconversion was documented. This case highlights that adult HAV can rarely present with polyserositis and acalculous cholecystitis yet resolve without invasive therapy, and that cholestatic flares and delayed serologic evolution may accompany otherwise benign convalescence.

## Introduction

Hepatitis A virus (HAV) is a non‑enveloped, positive‑sense RNA virus (*Picornaviridae*) transmitted predominantly by the fecal-oral route and remains a major cause of acute viral hepatitis globally, especially where sanitation is suboptimal. Most childhood infections are asymptomatic, whereas adults more often develop jaundice and symptomatic disease; nevertheless, the clinical course is typically self‑limited with lifelong immunity after infection [1,2]. Atypical adult presentations are increasingly recognized and include cholestatic and relapsing hepatitis, as well as extrahepatic manifestations such as serositis and acalculous cholecystitis (AAC) [1-4]. Polyserositis (e.g., ascites with pleural effusions) in HAV is uncommon but documented, generally transient, and thought to reflect immune‑mediated inflammation and capillary leak rather than progressive liver failure [3]. HAV‑associated AAC, gallbladder inflammation without gallstones, has been reported in adults and usually resolves with conservative therapy, avoiding cholecystectomy [4]. Serologically, anti‑HAV IgM typically appears near symptom onset and wanes over approximately three to six months (occasionally longer), while anti‑HAV IgG indicates past infection and persists for life [5,6]. Rationale for reporting: adult HAV with concurrent polyserositis and AAC is rare, and mid‑course cholestatic bilirubin rebound can be misinterpreted as decompensation. We report a young adult with HAV presenting with moderate ascites, bilateral pleural effusions, and imaging‑confirmed AAC, who developed a transient cholestatic bilirubin spike but recovered fully under conservative management, with IgM persistence and delayed IgG seroconversion consistent with expected serologic kinetics.

## Case presentation

A 20-year-old previously healthy male presented to our hospital with a 12-day history of intermittent fever with chills, central abdominal pain for seven days, and nausea with three to four episodes of non-bilious vomiting over the past four days. On admission, he was afebrile, conscious with a Glasgow Coma Scale (GCS) score of 15/15, and maintaining oxygen saturation on room air. His vitals showed pulse rate of 102 bpm, blood pressure of 110/70 mmHg, and respiratory rate of 20/min. General examination revealed icterus. Examination of the central nervous system, respiratory system, and cardiovascular system was largely normal. Abdominal examination showed fluid thrill with deep abdominal tenderness without guarding or rigidity. No organomegaly was palpable. Laboratory investigations revealed normal hemoglobin and leukocyte counts with thrombocytopenia. Liver function tests demonstrated marked transaminitis, elevated bilirubin, and raised alkaline phosphatase. Serum albumin was mildly reduced, with elevated serum lactate dehydrogenase (LDH) and C-reactive protein (CRP). The coagulation profile showed a significantly prolonged prothrombin time (PT) and international normalized ratio (INR) (Table 1).

**Table 1:** Basic blood profile. HCT: hematocrit; MCV: mean corpuscular volume; MCH: mean corpuscular hemoglobin; MCHC: mean corpuscular hemoglobin concentration; SGPT: serum glutamic pyruvic transaminase; ALT: alanine transaminase; SGOT: serum glutamic oxaloacetic transaminase; AST: aspartate aminotransferase; INR: international normalized ratio; LDH: lactate dehydrogenase; HIV: human immunodeficiency virus; HCV: hepatitis C virus.

Test	Day 1	Day 3	Day 5	Day 7	Day 9	Day 11	Day 13	Day 64	Day 90	Normal range
Hemoglobin (Hb)	14.8 g/dl	12.9 g/dl	14.2 g/dl	12.2 g/dl	11.1 g/dl	-	-	11.4 g/dl	-	10 - 16.5g/dl
Total leucocyte count (WBC)	5400 cells/cumm	5600 cells/cumm	4400 cells/cumm	4400 cells/cumm	5400 cells/cumm	-	-	4900 cells/cumm	-	4000-11000 cells/cumm
Neutrophils	58%	72%	73%	68%	64%	-	-	65%	-	40 - 75%
Lymphocyte	32%	22%	21%	26%	28%	-	-	27%	-	20 - 45%
Eosinophils	1%	1%	1%	1%	1%	-	-	4%	-	1 - 6%
Monocytes	9%	5%	5%	7%	7%	-	-	4%	-	2 - 8%
Platelet count	0.7 lakh	0.9 lakh	1.0 lakh	1.2 lakh	1.2 lakh	-	-	1.9 lakh	-	1.5 - 4.5 lakh
RBC	4.93 m/mm³	4.35 m/mm³	4.77 m/mm³	4.14 m/mm³	3.72 m/mm³	-	-	3.71 m/mm³	-	3.8 - 6.0 m/mm³
Hct	46.9%	42%	45.5%	39.2%	34.8%	-	-	35.0%	-	33 - 54%
MCV	95.1 ﬂ	96.6 ﬂ	95.4 ﬂ	94.7 ﬂ	93.4 ﬂ	-	-	93.2 ﬂ	-	80 - 100 ﬂ
MCH	30.0 pg	29.6 pg	29.9 pg	29.5 pg	29.8 pg	-	-	29.4 pg	-	25 - 32 pg
MCHC	31.6 g/dl	30.6 g/dl	31.3 g/dl	31.1 g/dl	31.9 g/dl	-	-	31.9 g/dl	-	28 - 36 g/dl
Reticulocyte count	1%	-	-	-	-	-	-	-	-	0.2 - 2.0%
Blood urea	25 mg/dl	27 mg/dl	24 mg/dl	14 mg/dl	-	-	-	-	-	Male: 19.0 - 42.8 mg/dl
Creatinine	0.8 mg/dl	0.8 mg/dl	0.7 mg/dl	0.5 mg/dl	-	-	-	-	-	Male: 0.66 -1.25 mg/ dL
Serum sodium	136 mmol/L	137 mmol/L	137 mmol/L	134 mmol/L	-	-	-	-	-	135 - 145 mmol/L
Serum potassium	4.5 mmol/L	4.7 mmol/L	4.9 mmol/L	4.5 mmol/L	-	-	-	-	-	3.5 - 5.5 mmol/L
Serum bilirubin (total)	7.4 mg/dl	8.3 mg/dl	7.5 mg/dl	6.8 mg/dl	7 mg/dl	6.6 mg/dl	7.4 mg/dl	1.1 mg/dl	0.8 mg/dl	0.2 - 1.3 mg/dl
Direct bilirubin	6 mg/dl	7.1 mg/dl	6.4 mg/dl	6 mg/dl	6.2 mg/dl	5.6 mg/dl	6.4 mg/dl	0.4 mg/dl	0.3 mg/dl	0.0 to 0.3 mg/dL
Indirect bilirubin	1.4 mg/dl	1.2 mg/dl	1.1 mg/dl	0.8 mg/dl	0.8 mg/dl	1 mg/dl	1 mg/dl	0.7 mg/dl	0.5 mg/dl	0.2 to 0.8 mg/dL
SGPT/ALT	3558 U/L	2350 U/L	1471 U/L	950 U/L	561 U/L	366 U/L	302 U/L	54 U/L	76 U/L	Male: <50 U/L
SGOT/AST	2315 U/L	1026 U/L	438 U/L	147 U/L	92 U/L	71 U/L	63 U/L	55 U/L	46 U/L	Male: 17-59 U/L
Serum alkaline phosphatase	201 U/L	152 U/L	158 U/L	185 U/L	227 U/L	256 U/L	261 U/L	134 U/L	119 U/L	38 - 126 U/L
Prothrombin time	33.7	22.6	17.7	14.8	14.1	-	11.5	-	-	9.8 - 12.1
INR	2.99	1.99	1.56	1.3	1.2	-	1.0	-	-	0.6 - 1.5
Serum calcium	9.4 mg/dl	-	-	8.8 mg/dl	-	10 mg/dl	-	-	-	8.6 - 10.2 mg/dl
Serum phosphorus	2.9 mg/dl	-	-	-	-	-	-	-	-	2.5 - 4.5 mg/dl
Total protein	6.3 g/dl	-	-	-	-	-	-	-	-	6.4 - 8.3 g/ dl
Serum LDH	982 U/L	-	-	-	-	-	-	-	-	120 - 246 U/L
Serum magnesium	2.2 mg/dl	-	-	-	-	-	-	-	-	1.6 - 2.3 mg/dl
Serum procalcitonin	1.74 ng/ ml	-	-	-	-	-	-	-	-	<0.500 ng/ml
Serum albumin	3.2 g/dl	-	-	-	-	-	-	-	-	3.5 - 5.2 g/dl
Serum amylase	35 U/L	-	36 U/L	-	-	-	-	-	-	30 - 110 U/L
Serum lipase	25 U/L	-	15 U/L	-	-	-	-	-	-	0 - 160 U/L
Serum ammonia	144 µmol/L	-	82 µmol/ L		64 µmol/ L	-	-	-	-	9 - 30 µmol/L
Serum C-reactive protein	51 mg/L	-	-	-	-	-	-	-	-	<10 mg/L

HAV IgM was positive, while hepatitis B virus (HBV), hepatitis C virus (HCV), and hepatitis E virus (HEV) serologies were negative (Table 2).

**Table 2:** Viral markers of the patient. HIV: human immunodeficiency virus; HCV: hepatitis C virus; HBsAg: hepatitis B surface antigen.

Test	Day 1	Day 5	Day 14	Day 90	Normal range
Hepatitis A antibody (anti-HAV) IgG	0.14	-	0.15	9.04	<0.90 negative; 0.90-1.0 equivocal; >1.0 positive
Hepatitis A antibody (anti-HAV) IgM	6.33	5.93	2.19	2.46	<0.90 negative; 0.90-1.0 equivocal; >1.0 positive
Hepatitis E virus - IgG (HEV IgG)	0.57	-	-	-	<1.0 negative; >1.0 positive
Hepatitis E virus - IgM (HEV IgM)	0.13	-	-	-	<1.0 negative; >1.0 positive
HIV	Non-reactive	-	-	-	Non-reactive
HCV	Non-reactive	-	-	-	Non-reactive
HBsAg	Non-reactive	-	-	-	Non-reactive

**Figure 1:**
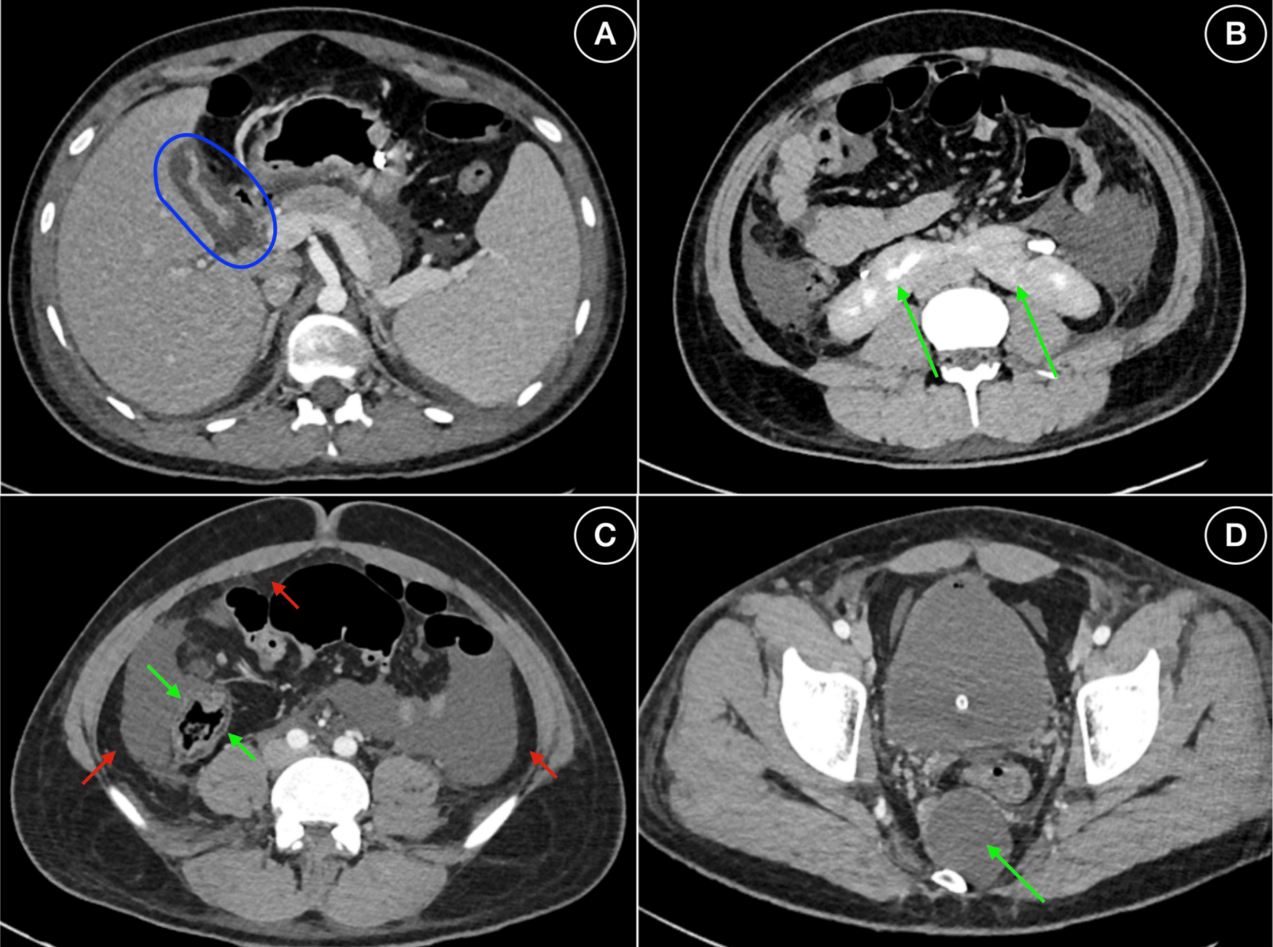
Contrast-enhanced computed tomography of the whole abdomen. (A) Contracted gallbladder with surrounding pericholecystic fluid suggestive of acalculous cholecystitis (blue oval). (B) Lower poles of kidneys fused in the midline by a membranous isthmus forming a parenchymal bridge at the L3 vertebral level, consistent with horseshoe kidneys (green arrows). (C) Edematous thickening of the ascending colon, indicating inflammatory/infective pathology (green arrows), with associated ascites visible (red arrows). (D) A well-defined, rounded, hypodense benign lesion measuring 4.4 × 4.2 × 4.3 cm in the sacral region posterior to the rectum, suggestive of a tail-gut cyst (green arrow).

Venous blood gas indicated mild lactic acidosis with normal pH and bicarbonate levels. Chest X-ray showed bilateral pleural effusion. Ultrasound showed hepatosplenomegaly (liver = 17.3 cm, spleen = 15.9 cm), moderate ascites, bilateral mild pleural effusions, and a contracted gallbladder with pericholecystic fluid but no calculi. Contrast-enhanced computed tomography (CECT) of the abdomen confirmed the same findings along with mild wall thickening of the cecum, suggestive of inflammation and an incidental tailgut cyst. Kidneys were fused at the lower poles (horseshoe kidneys) with a right renal cortical cyst (Figure 1).

Ascitic fluid analysis revealed yellow alkaline fluid with a low serum ascites albumin gradient (SAAG) and high protein, indicating exudative etiology with high protein content (Table 3).

**Table 3:** Lipid profile, ascitic fluid analysis, urine, and stool investigations. HDL: high-density lipoprotein; VLDL: very-low-density lipoprotein; LDL: low-density lipoprotein; TLC: total lymphocyte count; LDH: lactate dehydrogenase; ADA: adenosine deaminase; RBC: red blood cells.

Test	Day 1	Day 3	Day 5	Day 7	Day 9	Normal range
Serum	63 mg/dl	-	-	-	-	Borderline high: 200 - 239 mg/dL
Serum triglycerides	218 mg/dl	-	-	-	-	<161.0 mg/dl
Serum HDL cholesterol	16 mg/dl	-	-	-	-	Male: 35.3 - 79.3 mg/dl
Serum VLDL cholesterol	44 mg/dl	-	-	-	-	2 - 30 mg/dl
Direct LDL	<30 mg/dl	-	-	-	-	<= 100 mg/dl
Ascitic fluid TLC	350 cells/cum	-	-	-	-	<250 cells/cum
Ascitic fluid polymorphs	20%	-	-	-	-	-
Ascitic fluid lymphocytes	80%	-	-	-	-	-
Ascitic fluid protein	3.7 gm%	-	-	-	-	<2.5 gm%
Ascitic fluid sugars	103 mg%	-	-	-	-	70-106 mg/dl
Ascitic fluid albumin	1.6 gm%	-	-	-	-	<2.5 gm%
Ascitic fluid LDH	99 IU/L	-	-	-	-	<225 IU/ L
Ascitic fluid ADA	12.98 IU/L	-	-	-	-	<30 IU/L
Urine protein	+++	-	+	-	Traces	Nil
Urine epithelial cells	2-4	-	4-6	-	2-4	Nil
Urine pus cells	6-8	-	1-2	-	4-6	Nil
Urine RBC	6-8	-	None	-	1-2	Nil
Urine sugars	++	-	None	-	None	Nil
Urine cast/crystals	None	-	None	-	None	Nil
Urine culture	Sterile	-	-	-	-	Sterile
Stool for occult blood	Negative	-	-	-	-	Negative

Gram stain and acid-fast bacilli (AFB) smear were negative, which led us to suspect viral peritonitis. The patient was managed conservatively. Over the course of the treatment, his distension of the abdomen decreased, and shifting dullness and tenderness were reduced markedly on abdominal examination. He received intravenous ceftriaxone 1 g every 12 hours for seven days and intravenous metronidazole with a 15 mg/kg loading dose followed by 7.5 mg/kg every six hours for 10 days. Hepatoprotective treatment included ursodeoxycholic acid, silymarin, vitamin K, and supportive care. Oral intake was gradually reintroduced after 48 hours of nil per os. Over the next 10 days, his liver function tests showed progressive improvement. Repeat X-ray of the chest on discharge showed complete resolution of pleural effusion.

By discharge on day 13, bilirubin had decreased and remained elevated, but transaminitis showed a significant resolution trend with resolution of coagulopathy (Table 1). Throughout admission, the patient remained hemodynamically stable, without encephalopathy, and his platelet count recovered in parallel with the complete resolution of symptoms. At three-month follow-up, liver biochemistry had normalized; serology showed persistent HAV-IgM reactivity with seroconversion to HAV-IgG, a pattern consistent with convalescent-phase immunity.

## Discussion

Most adult HAV infections are self-limited, with severe disease and extrahepatic complications being uncommon. Recognized “atypical” courses include prolonged cholestasis, relapsing hepatitis, and selected extrahepatic syndromes; these patterns are increasingly described but remain uncommon relative to the classic, monophasic illness [7].

We managed our patient as HAV-associated acute liver injury (ALI) rather than acute liver failure (ALF) because, despite coagulopathy, the patient never developed hepatic encephalopathy (HE). This distinction matters clinically and terminologically: contemporary guidance defines ALF by the presence of HE within a specified interval after jaundice, whereas ALI denotes coagulopathy without encephalopathy. Framing our case as HAV-ALI, therefore, aligns with the European Association for the Study of the Liver (EASL) guidance and recent hepatology reviews, which emphasize encephalopathy as the linchpin of the ALF definition [8,9].

Our patient’s serologic trajectory also tracked closely with canonical kinetics. By the three-month visit, he remained anti-HAV IgM-positive, had seroconverted to IgG, and had biochemical resolution. This pattern fits public health and hepatology references showing that IgM typically becomes detectable days to weeks before symptom onset and usually persists for up to approximately six months, while IgG appears soon thereafter and persists for life [10]. Importantly, the literature documents longer persistence of low-titer IgM in a subset, sometimes beyond six months, and highlights that apparent late positivity can occasionally be false-positive or related to autoimmune phenomena; these reports help clinicians avoid over-interpreting isolated IgM results in convalescence [5,11]. When clinical enzymes and course normalize (as in our case), no additional HAV-directed therapy is indicated, but in any future discordance between serology and biochemistry, HAV RNA by reverse-transcription polymerase chain reaction (RT-PCR) can adjudicate true ongoing infection versus residual/false-positive IgM [10,12,13].

The clinical course was likewise typical for adult HAV: most adults recover within two months, though ~10-15% have prolonged or relapsing symptoms for as long as six months. Our patient showed a steady, uncomplicated recovery without relapse, matching the common self-limited pattern [10]. We remained attentive to the cholestatic variant, given its potential for pruritus and protracted jaundice. Evidence for specific treatment is limited to case series and case reports, but where cholestasis is severe and persistent, short corticosteroid courses have been associated with symptomatic improvement; in our case, conservative management sufficed and steroids were unnecessary [14].

Two atypical/extrahepatic features deserve comment. First, the patient had serosal involvement with ascites (and no alternative cardiorenal or portal-hypertensive driver). Polyserositis with HAV is better described in pediatrics but is uncommon in adults; published reports (including pleural effusion with ascites) generally describe a self-resolving course with supportive care, mirroring our experience [15,16]. Second, the patient had imaging-proven acute acalculous cholecystitis (AAC), a recognized but uncommon HAV association in adults, again remitting without surgery as the hepatitis improved, consistent with adult case literature [17]. These manifestations broaden the adult phenotype and help explain the patient’s early abdominal symptoms without invoking alternative biliary etiologies.

Taken together, this case highlights three practical lessons in adult hepatitis A. First, in the absence of encephalopathy, we describe the presentation as acute hepatitis A with coagulopathy (consistent with contemporary criteria and avoiding overcalling ALF), which clarifies prognosis without implying a different therapeutic pathway. Second, persistence of anti-HAV IgM at around three months alongside IgG seroconversion and normalized biochemistry matches expected serologic kinetics and favors reassurance over repeated testing or intervention. Third, extrahepatic serositis and acalculous cholecystitis, while uncommon in adults, can accompany HAV and typically resolve with supportive care. These points reinforce disciplined nomenclature, measured interpretation of serology, and conservative management of atypical features.

## Conclusions

HAV-associated ALI (coagulopathy without encephalopathy) can resolve completely with supportive care. Precise classification, awareness of serologic kinetics, and the usually self-limited nature of extrahepatic features help avoid unnecessary interventions and guide focused follow-up.
